# Skeletal muscle and fiber type-specific intramyocellular lipid accumulation in obese mice

**DOI:** 10.17305/bjbms.2021.5876

**Published:** 2021-12

**Authors:** Nejc Umek, Simon Horvat, Erika Cvetko

**Affiliations:** 1 Institute of Anatomy, Faculty of Medicine, University of Ljubljana, Ljubljana, Slovenia; 2 Department of Animal Science, Chair for Genetics, Biotechnology and Immunology, Biotechnical Faculty, University of Ljubljana, Domžale, Slovenia

**Keywords:** Skeletal muscle, lipid accumulation, fiber type, obesity, insulin resistance

## Abstract

In obesity, accumulation of lipid droplets in skeletal muscle fibers and a shift towards fast muscle fiber types can both contribute to insulin resistance. However, it is not yet clear how intramyocellular lipid accumulation and fiber type changes are associated. Therefore, we investigated to what extent the lipids accumulated in a fiber type-specific manner in the functionally similar fast-, intermediate- and slow-twitch gastrocnemius, plantaris, and soleus muscles, respectively, in high-fat diet-induced obese 54-week-old female C57BL/6JOlaHsd mice (n = 9) compared to control standard-diet-treated lean mice (n = 9). A high-fat diet was administered for 26 weeks. Fiber-type specific intramyocellular lipid content analysis and muscle fiber typing were performed using histochemical analysis of lipids with Sudan Black and immunohistochemical analysis of myosin heavy chains on serial sections of skeletal muscles. Compared to the lean mice, the lipid accumulation was most prominent in types 2a and 2x/d fibers (p < 0.05) of fast-twitch gastrocnemius and intermediate plantaris muscles in the obese mice, while in slow-twitch soleus muscle, there was no significant lipid accumulation in the obese animals. Furthermore, the slow-twitch soleus muscle of the obese mice with no significant change in muscle fiber diameters exhibited the most pronounced shift towards fast-type myosin heavy chain isoform expression (p < 0.05). In contrast, the fast-twitch and intermediate-twitch gastrocnemius and plantaris muscles, respectively, in which the muscle fiber diameters increased (p < 0.05), were more resistant toward myosin heavy chain expression changes. In conclusion, we demonstrated both muscle- and fiber-type specificity in intramyocellular lipid accumulation in obese mice, suggesting that in obesity, similar muscle fiber types in different muscles accumulate lipids differentially.

## INTRODUCTION

Glucose metabolism disorders are among the most common metabolic disorders in the developed world. In 2019, the global prevalence estimates of metabolic syndrome and diabetes were over one billion and 463 million, respectively [1]. The skeletal muscle accounts for 40-50% of the body weight and mediates 75% of insulin-stimulated glucose uptake, and is therefore one of the main potential targets for the treatment of metabolic syndrome, diabetes, and many other disorders [2-4]. Each skeletal muscle has different fiber type composition. Muscle fibers are classified on the basis of the expression of different myosin heavy chain isoforms, with each type displaying particular biochemical and physiological properties [5]. Skeletal muscle type 1 fibers (slow-twitch oxidative fibers) have a high content of glucose transporter 4 (GLUT4) and are highly insulin sensitive, in contrast to type 2b fibers (fast-twitch glycolytic fibers) which have a low content of GLUT4 and are poorly insulin sensitive. Types 2a and 2x/d fibers show intermediate insulin sensitivity and express intermediate levels of GLUT4 [6,7]. Fiber-type shift toward fast, less insulin-sensitive types has been implicated in the pathogenesis of insulin resistance in obesity and type 2 diabetes since a decreased proportion of type 1 fibers has been noted in insulin-resistant states such as obesity, metabolic syndrome, and prolonged bed-confinement [8-10]. However, studies in type 2 diabetic patients have not been very consistent. While some studies noted a decreased proportion of type 1 muscle fibers [8,10-12], others found no changes in the skeletal muscle fiber distribution [13,14].

In skeletal muscles, lipid molecules are stored both extramyocellularly (in the connective tissue between muscle fibres) and intramyocellularly (as lipid droplets within skeletal muscle fibers) [15]. The intramyocellular lipids (IMCLs), composed mainly of triglycerides, are known to be metabolically active, providing an important energy source for skeletal muscle cells during exercise. In obesity, the IMCL content is increased, and such an increase has been correlated with insulin resistance since lipid derivatives such as diacylglycerol and ceramide can mediate insulin resistance [16]. Chronic lipid accumulation in muscles in prolonged obesity has also been associated with other adverse metabolic effects, including mitochondrial dysfunction and decreased muscle protein synthesis through lipotoxic interference with amino acid incorporation in muscle proteins [17].

The IMCL accumulation has been shown to differ according to skeletal muscle typology. During short term (24 hours) high-fat diet treatment in mice, the slow-twitch soleus muscle had higher IMCL content than the fast-twitch extensor digitorum longus muscle owing to the higher population of type 2a fibers [18]. In contrast, both in rats and mice, the fast-twitch tibialis anterior and extensor digitorum muscles showed significantly higher IMCL content than the slow-twitch soleus and diaphragm muscles during the long-term (16-24 weeks) high-fat diet-induced obesity trials [17,19]. The inconsistencies could have resulted from the differential time exposure to high-fat diet and obesity, age of animals, different animal models, different rate of IMCL accumulation, different IMCL accumulation capacity, or the use of muscles with different functions (soleus, diaphragm, tibialis anterior, and extensor digitorum longus). Therefore, we aimed to determine the changes in myosin heavy chain expression and fiber-type specific IMCL content in high-fat diet-induced obese mice, using three functionally similar muscles: The fast-, intermediate- and slow-twitch gastrocnemius, plantaris, and soleus muscles, respectively.

## MATERIALS AND METHODS

All study protocols were assessed and approved by the Ethical Committee for laboratory animals of the Republic of Slovenia (Permit numbers: U34401-34/2013/6, U34401-34/2014/9). The study was performed in full compliance with the European Union legislation on the protection of animals used for scientific purposes (Directive 2010/63/EU) and in accordance with the recommendations of the National Institutes of Health’s Guide for the Care and the Use of Laboratory Animals and the ARRIVE guidelines.

### Animals and diets

The study animals consisted of 18-week-old female C57BL/6JOlaHsd mice obtained from the Harlan Laboratories – Envigo (Italy). Following the 3R principles (replacement, reduction, and refinement), this study was performed in mice that were primarily included in the study of changes in skeletal muscle capillarization during obesity and insulin resistance [20]. The animals were reared in individually ventilated cage systems at the Centre for Laboratory Animals of the Biotechnical Faculty of the University of Ljubljana, Slovenia. The rearing environmental conditions were as follows: Temperature maintained at 23 ± 1°C; humidity at 40-60%; with 12-hour light/dark cycle, and water and food available *ad libitum*. After quarantine and acclimatization period, at the age of 28 weeks, the animals were randomly assigned to two study groups: The high-fat diet-induced obese group (n = 9) and the control-diet treated lean group (n = 9). The former was fed with a purified high-fat diet for 26 weeks (D12108C, Research Diets, Inc., New Brunswick, NJ, USA), while the latter received a standard maintenance diet (4RF18, Mucedola, Milan, Italy).

At the age of 54 weeks, the animals were sacrificed by cervical dislocation and weighed, following which the gastrocnemius, soleus, and plantaris muscles were harvested from the left hind limbs and frozen in liquid nitrogen and preserved−80°C until analysis.

### Oral glucose tolerance test (OGTT)

The status of tolerance for glucose was assessed by the OGTT: A 25% glucose solution (2 g kg^−1^) was administered via orogastric tube feeding following 6-hour fasting (water available *ad libitum*) [21], with venous blood glucose assayed before and at 15, 30, 60, and 120 minutes after the glucose administration using the Bayer Contour glucose meter (Ascensia Diabetes Care Holdings AG, Basel, Switzerland). Blood samples were collected from the tail vein of the animals.

### Histological analysis

For histological analysis transverse, 10 mm thick cryosections of the skeletal muscles cut with the cryomicrotome Leica CM 1950 (Leica Microsystems, Germany) were stained with hematoxylin and eosin (Sigma-Aldrich Corp, St. Louis, MO, USA) as described by Wang et al. [22]. The skeletal muscle sections were analyzed for signs of atrophy, necrosis, regeneration, or inflammation by a trained evaluator blinded to group assignment [23].

### Myosin heavy chain immunohistochemical staining

Serial transverse 10 mm thick cryosections were marked with three monoclonal antibodies (BA-D5, BF-F3, and SC71) purchased from Deutsche Sammlung von Mikroorganismen und Zellkulturen DSMZ, Braunschweig, Germany and a fourth (6H1) sourced from the Developmental Studies Hybridoma Bank, Iowa City, USA[24,25]. The antibodies were immunoreactive to specific myosin heavy chain isoforms: BA-D5 antibodies for myosin heavy chain-1; BF-F3 for myosin heavy chain-2b; SC71 for myosin heavy chain-2a; and 6H1 for myosin heavy chain-2x/d. All antibody applications were at 1:100 dilutions, except for BF-F3 at 1:30. Myosin heavy chain isoforms 1, 2a, and 2b were revealed by a horseradish peroxidase-conjugated secondary antibody P0260 (Dako, Glostrup, Denmark), while 2x/d was revealed by a Polymer Detection System (Novolink, Leica Biosystems, Newcastle, UK). The expression of myosin heavy chain isoforms on serial sections by the indirect immunoperoxidase method was used for muscle fiber phenotyping, while the specificity of immunostaining was confirmed by lack of immunoreactivity in sections incubated without primary antibodies [26-28].

### Lipid histochemical staining

Lipid content analysis was adopted from Masgrau et al. [17]. Lipid staining of 10 mm thick cryosections was performed with Sudan Black B (Sigma-Aldrich Corp, St. Louis, MO, USA) which stains neutral lipids with a blue-black tint. Stock solution was prepared by diluting the powder in 70% ethanol until saturation, stirred for 1 hour at 60°C, and cooled down to room temperature at least 1 day before application. Just before staining, the stock solution was filtered by grade 589/1 black ribbon filter paper (Sigma-Aldrich Corp, St. Louis, MO, USA). Serial sections were then immersed in a working solution of Sudan Black for 60 minutes, after rinsing in 70% ethanol for 10 seconds. After incubation, sections were rinsed in tap water and covered-slipped using glycerol gelatine.

### Image acquisition and data analysis

Image capture was performed with a Nikon Eclipse 8000 microscope (×20 objective, numerical aperture: 0.50) equipped with a Nikon digitalized camera DXM 1200F and computer software for image acquisition (Lucia GF software, version 4.82, Laboratory imaging, Prague, Czech Republic). Serial sections stained by the myosin heavy chain isoforms-specific monoclonal antibodies and Sudan Black dye were captured at 2560 × 1920-pixel resolution, with a minimum of three fields of view randomly sampled for each muscle. Each muscle fiber was delineated by the Ellipse image analysis software (ViDiTo, Kosice, Slovakia). The classification of the muscle fibers and estimation of their average diameter and numerical proportion was performed with the software for muscle fiber type classification and analysis by Karen et al. [27]. On average, 120 fibers per skeletal muscle were analyzed. The muscle fiber diameter was estimated using minimal Ferret diameter [29]. Lipid content analysis was performed using the Ellipse software. First, the area of muscle fiber occupied by lipids was determined by thresholding. Then, the lipid content index was calculated as 100 times the ratio of cross-sectional area of muscle fiber occupied by lipid droplets to cross-sectional area of muscle fiber. The average lipid content index was calculated for each muscle fiber type in every studied skeletal muscle [17,30]. All study protocols (muscle sectioning, staining, and image analysis) were executed in a blinded manner throughout the study.

### Statistical analysis

The GraphPad Prism 8 (GraphPad Software, San Diego CA, USA) was utilized for all statistical analyses and graphing. The normality of continuous data was analyzed with the Shapiro-Wilk test. The Student’s *t*-test or Mann–Whitney U test was applied for comparison between two study groups. A repeated-measures ANOVA with Sidak correction was used for comparison of serial glucose measurements during OGTT. For comparison of numerical proportions, diameters, and lipid content index for each fiber type, a repeated-measures three-way ANOVA with Sidak *post hoc* tests was used. The association between lipid content index and muscle fiber diameter within the same fiber type was assessed using the Pearson correlation coefficient. Data are presented as means ± standard deviations and statistical difference was considered significant at *p* < 0.05.

## RESULTS

**FIGURE 1:**
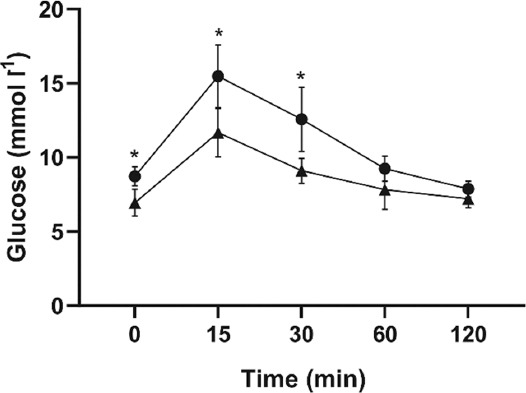
Oral glucose tolerance test in high-fat diet-induced obese (•) (n=9) and lean (▲) (n=9) mice. Data are means and SD. *p<0.05 versus lean mice.

Mice on a high-fat diet had a higher body mass (36.9 ± 2.2 g vs. 27.1 ± 3.0 g, *p* < 0.0001) and basal hyperglycemia and decreased tolerance for glucose compared to the standard-diet treated lean mice in a standard OGTT (Figure 1).

**FIGURE 2:**
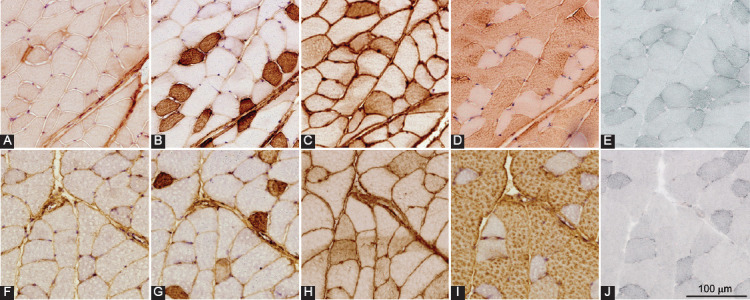
Fiber type-specific intramyocellular lipid content of gastrocnemius muscle. Expression of myosin heavy chain isoforms 1 (A and F), 2a (B and G), 2x/d (C and H), 2b (D and I), and Sudan Black staining (E and J) in successive cross-sections of gastrocnemius muscle of high-fat diet-induced obese (A-E) and lean (F-J) mice.

**FIGURE 3:**
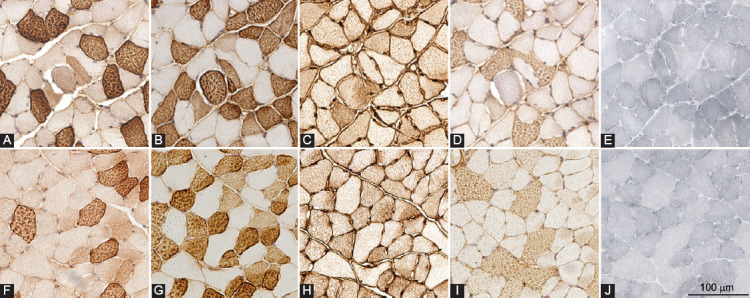
Fiber type-specific intramyocellular lipid content of plantaris muscle. Expression of myosin heavy chain isoforms 1 (A and F), 2a (B and G), 2x/d (C and H), 2b (D and I), and Sudan Black staining (E and J) in successive cross-sections of plantaris muscle of high-fat diet-induced obese (A-E) and lean (F-J) mice.

**FIGURE 4:**
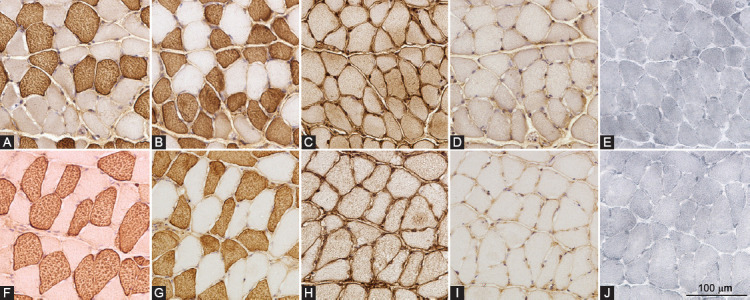
Fiber type-specific intramyocellular lipid content of soleus muscle. Expression of myosin heavy chain isoforms 1 (A and F), 2a (B and G), 2x/d (C and H), 2b (D and I), and Sudan Black staining (E and J) in successive cross-sections of soleus muscle of high-fat diet-induced obese (A-E) and lean (F-J) mice.

**FIGURE 5:**
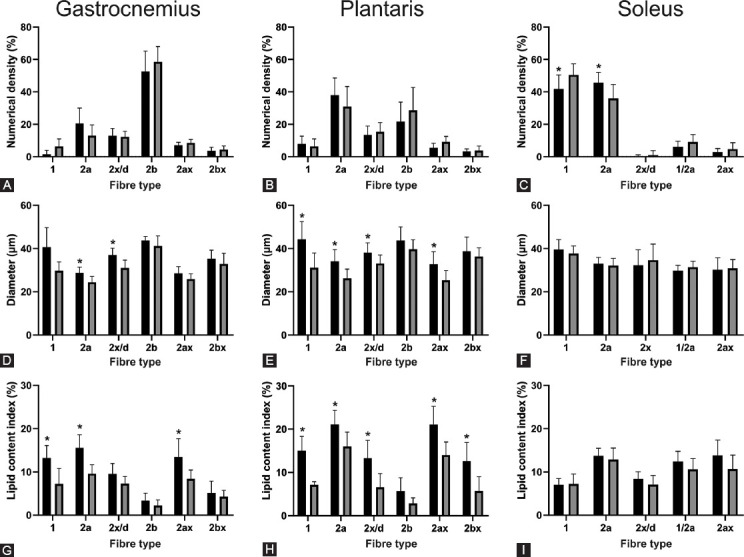
Fiber-type specific muscle fiber numerical density (A-C), diameter (D-F), and lipid content index (G-I) in gastrocnemius (A, D, G), plantaris (B, E, H), and soleus (C, F, I) muscles of high-fat diet-induced obese (■) (n=9) and lean (■) mice (n=9). Data are means and SD. *p<0.05 versus lean mice.

Typical staining patterns for myosin heavy chains and Sudan Black in the gastrocnemius, plantaris, and soleus muscles of obese and lean mice are shown in Figures 2-4, respectively. A comparison of muscle phenotype parameters such as numerical density, diameter, and lipid content index for different muscle fiber types between obese and lean mice and three skeletal muscles is displayed in Figure 5.

The repeated-measures three-way ANOVA revealed that there was a statistically significant interaction between the effects of the study group (obese and lean mice) and skeletal muscle (gastrocnemius, plantaris, and soleus muscles) for lipid content index (*p* = 0.009) and muscle fiber diameter (*p* = 0.044), suggesting that different skeletal muscles responded differently to high-fat diet treatment. Moreover, there was also a statistically significant interaction between the effects of the study group (obese and lean mice) and muscle fiber type (type 1, 2a, 2x/d, 2b, and hybrid fibers) for lipid content index (*p* = 0.015) and muscle fiber diameter (*p* = 0.038), suggesting that different fiber types responded differently to high-fat diet treatment. There was no significant correlation between muscle fiber diameter and lipid content index within the same fiber type in any skeletal muscle.

The gastrocnemius muscle was composed primarily of type 2b muscle fibers with no significant differences in the numerical densities of different fiber types between the obese and lean mice. The diameters (Figure 5A, 5D, 5G) of types 2a (*p* = 0.003) and 2x/d (*p* = 0.027) fibers of gastrocnemius muscle in the obese mice were significantly larger compared to the lean mice; and the lipid content index was significantly higher in type 1 (*p* = 0.001), 2a (*p =* 0.002), and 2ax fibers (*p* = 0.007) of the obese compared to lean mice.

The plantaris muscle was composed mainly of types 2a, 2b, and 2x/d muscle fibers, with no significant differences in the numerical densities of different fiber types between the obese and lean mice (Figure 5B, 5E, 5H). The diameters of types 1 (*p* = 0.003), 2a (*p* = 0.003), 2x/d (*p* = 0.027), and 2ax (*p* = 0.029) fibers of plantaris muscle in the obese mice were significantly larger compared to the lean mice; and the lipid content index was significantly higher in type 1 (*p* = 0.008), 2a (*p* = 0.008), 2x/d (*p* = 0.032), 2ax (*p* = 0.001), and 2bx fibers (*p* = 0.040) of the obese compared to lean mice.

The soleus muscle was composed mainly of types 1 and 2a muscle fibers, with a very low numerical density of types 2x/d and no 2b fibers (Figure 5C, 5F, 5I). Compared to the lean mice, the numerical densities of type 1 were significantly lower (*p* = 0.042), whereas of type 2a were significantly higher (*p* = 0.024) in obese mice. In soleus muscle, there were no significant differences in muscle fiber diameter and lipid content index between study groups.

**FIGURE 6:**
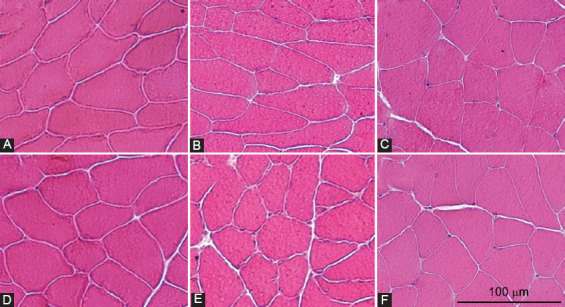
Representative images of hematoxylin and eosin-stained cross-sections of gastrocnemius (A and D), plantaris (B and E), and soleus (C and F) muscles of high-fat diet-induced obese (A-C) and lean (D-F) mice.

Muscle fibers of all three skeletal muscles in both obese and lean mice were arranged mosaically, meaning that no muscle fiber was completely surrounded by fibers of the same type. There were also no histologic signs of atrophy, necrosis, regeneration, or reparation such as centrally located nuclei, vesicular nuclei, basophilic muscle fibers, or abundant extracellular matrix deposits in any skeletal muscle (Figure 6).

Therefore, the most significant differences between obese and lean mice were found for diameter and lipid content index in gastrocnemius and plantaris muscles, with higher values always found in obese than lean mice.

## DISCUSSION

We found that 26 weeks of a high-fat diet resulted not only in basal hyperglycemia and decreased tolerance for glucose but also in an increase in muscle fiber size and IMCL content, particularly in type 2a and 2x/d muscle fibers, and more so in fast-twitch than slow-twitch skeletal muscles. Furthermore, in obese mice, the shift toward fast-type myosin heavy chain isoform expression was most pronounced in slow-twitch soleus muscle.

Slow-twitch muscles were shown to have higher IMCL content than fast-twitch muscles after short-term (24 hours) high-fat diet treatment in mice [18]. In contrast, and consistent with our findings, during long-term high-fat diet treatment in adult male rats and female mice, the IMCL content was significantly higher in fast-twitch muscles than in slow-twitch muscles [17,19]. However, in these investigations, antagonistic muscles were compared, which may influence the IMCL content due to the potential differential effect of increased body weight on skeletal muscle load during long-term high-fat diet treatment protocols [31]. Please note that some studies used optical density measures instead of lipid content index as an estimate of IMCL content, which could cause some discrepancies in the results [19]. Our results reaffirm that in obesity, the IMCL accumulation is skeletal muscle-specific, with greater prominence in fast-twitch muscles.

Komiya et al. demonstrated increased IMCL accumulation in 2a and 2x/d fibers, but not in type 1 or type 2b fibers, following short-term high-fat diet treatment in male mice [18]. In contrast to humans, in mice, type 1 fibers are believed to be less metabolically active than types 2a, which is supported by the observations that in mice and rats, succinate dehydrogenase (SDH) activity is the highest in type 2a muscle fibers [19,32]. We noted that during our long-term high-fat diet treatment, the IMCL accumulated in types 2a, 2x/d, and type 1 muscle fibers of fast-twitch muscles. Moreover, the IMCL content was highest in type 2a fibers, supporting the notion that in mice, type 2a fibers are the most oxidative. This is further supported by the observation that type 2a fibers have the smallest diameter because there is an inverse correlation between muscle fiber diameter and oxidative capacity, with small muscle fibers having higher oxidative enzyme activity and mitochondrial density than large fibers irrespective of their fiber types [20,33].

The shift toward fast-type myosin heavy chain isoform expression was most pronounced in the slow-twitch soleus muscle, in contrast to the fast-twitch gastrocnemius and intermediate plantaris muscles where no significant difference in the numerical density of different fibers types was demonstrated in the obese and lean mice. Similarly, several studies have also indicated that in obesity with insulin resistance, the shift toward fast-type myosin heavy chain isoforms expression was most pronounced in slow-twitch muscles and absent in fast-twitch muscles [34,35]. In our previous study, we also noted a shift toward fast-type myosin heavy chain isoform expression in non-weight bearing fast-twitch gluteus maximus muscles of the same mice used in the present study [20]. This suggests that in obesity with insulin resistance, there is likely a shift toward fast-type myosin heavy chain isoforms in both slow- and fast-twitch muscles and that increased weight-bearing probably protects the fast-twitch muscles from fiber type shifting. This is further supported by the observation that fast-twitch and intermediate weight-bearing muscles of the obese mice exhibited signs of hypertrophy due to increased body mass and muscle load, while in non-weight bearing fast-twitch gluteus muscle, there was no evidence of hypertrophy [20]. Note that the muscle fiber diameter changes could also arise from changes in physical activity, which was not monitored in our study. Interestingly, there were no signs of hypertrophy in soleus muscle, suggesting that slow-twitch muscles, although weight-bearing, are probably more resistant to the effect of increased load.

In the obese mice, no morphologic signs of atrophy, necrosis, or regeneration were found, suggesting that the basal hyperglycemia and decreased tolerance for glucose were not so severe to be toxic to the skeletal muscles. This is also congruent with the observations in diabetic patients, where diabetic myopathy is a rare complication found mostly in the late course of the disease, usually in association with poor glycemic control and other microvascular complications [36,37].

For the mouse line used in the present study, high-fat diet treatment produced the expected glycemic and body mass changes, including the establishment of insulin resistance [38]. High-fat diet-induced obesity and insulin resistance in mice is believed to be one of the best murine models to study type 2 diabetes. Other mice models, such as *ob/ob* mice (leptin mutant) and *db/db* mice (leptin receptor mutant) are also commonly used as preclinical models of type 2 diabetes. They develop hyperglycemia, obesity, and diabetes early and in severe form [39,40]. A significant lower proportion of type 2b fibers and smaller muscle diameter, muscle mass, and fiber grouping was noted in *db/db* mice [41,42]. Such early and significant muscle loss and atrophy are believed to be due to the presence of severe insulin resistance, hyperglycemia, loss of leptin signaling, metabolic derangements, and their consequent toxic effects on muscle growth and development. Hence, the muscle phenotype of *db/db* and *ob/ob* diabetic mice is probably not representative of the human muscle in the common form of adult-onset type 2 diabetes but rather of a rare subset of human morbid obesity due to lack of function of leptin and its receptor [43,44]. TallyHo mouse, as a polygenic model of adult-onset type 2 diabetes, has been proposed as a better preclinical model for disease-related skeletal muscle research; however, the lack of non-diabetic controls of the same strain is a major limitation of this model [43,45].

The high-fat diet-induced obese mice model used in our study is the most widely used model to study late-onset polygenic obesity-induced diabetes [46]. However, as any other rodent model, it has its limitations in recapitulating all the hallmarks of the complex disease in humans [47]. Our study was also performed only in female mice, and given that sex differences in insulin sensitivity have been reported in rat and mouse models, it would be important in future experiments to also explore the same study design in male mice [48-50].

Despite having the same polygenic background to obesity and diabetes development as in humans, the high-fat diet-induced obese mice model used in the present study exhibits relatively moderate basal hyperglycemia and decreased tolerance for glucose [51]. In this sense, this model recapitulates more of a pre-diabetic state than advanced diabetes with associated neuropathy, nephropathy, and liver steatosis [51]. On the other hand, our results showing significant changes in muscle lipid phenotype in such a pre-diabetic state can be regarded informative in that they demonstrate that the process of IMCL accumulation develops early on, even in a pre-diabetic state. Similarly, in insulin-resistant offspring of patients with type 2 diabetes, dysregulation of myocellular fatty acid metabolism was also detected, possibly due to mitochondrial dysfunction [52]. As the mouse models have an advantage over human studies in allowing vast tools of genetic and environmental manipulations, an extension of our study examining the mechanism and time-course by which IMCL accumulation contributes to the development of insulin resistance may further illuminate its etiologic significance.

## CONCLUSION

We demonstrated both muscle- and fiber-type specificity in IMCL accumulation in high-fat diet-induced obese mice. The IMCL accumulation was pronounced particularly in type 2a and 2x/d muscle fibers, and more so in fast-twitch gastrocnemius and plantaris muscles than in slow-twitch soleus muscles, suggesting that in obesity, similar muscle fiber types in different muscles accumulate lipids differentially.
